# Reporting of sex as a variable in cardiovascular studies using cultured cells

**DOI:** 10.1186/2042-6410-2-11

**Published:** 2011-11-07

**Authors:** K Efua Taylor, Catalina Vallejo-Giraldo, Niccole S Schaible, Rosita Zakeri, Virginia M Miller

**Affiliations:** 1 Department of Physiology and Biomedical Engineering, Mayo Clinic, 200 1st St SW, Rochester, MN 55905 USA; 2 Department of General Internal Medicine, Division of Cardiovascular Diseases, Mayo Clinic, 200 1st St SW, Rochester, MN 55905 USA; 3 Departments of Surgery, Physiology and Biomedical Engineering, Mayo Clinic, 200 1st St SW, Rochester, MN 55905 USA

## Abstract

**Background:**

Chromosomal complement, including that provided by the sex chromosomes, influences expression of proteins and molecular signaling in every cell. However, less than 50% of the scientific studies published in 2009 using experimental animals reported sex as a biological variable. Because every cell has a sex, we conducted a literature review to determine the extent to which sex is reported as a variable in cardiovascular studies on cultured cells.

**Methods:**

Articles from 10 cardiovascular journals with high impact factors (*Circulation*, *J Am Coll Cardiol*, *Eur Heart J*, *Circ Res*, *Arterioscler Thromb Vasc Biol*, *Cardiovasc Res*, *J Mol Cell Cardiol*, *Am J Physiol Heart Circ Physiol*, *J Heart Lung Transplant and J Cardiovasc Pharmacol*) and published in 2010 were searched using terms 'cultured' and 'cells' in any order to determine if the sex of those cells was reported. Studies using established cell lines were excluded.

**Results:**

Using two separate search strategies, we found that only 25 of 90 articles (28%) and 20 of 101 articles (19.8%) reported the sex of cells. Of those reporting the sex of cells, most (68.9%; n = 31) used only male cells and none used exclusively female cells. In studies reporting the sex of cells of cardiovascular origin, 40% used vascular smooth-muscle cells, and 30% used stem/progenitor cells. In studies using cells of human origin, 35% did not report the sex of those cells. None of the studies using neonatal cardiac myocytes reported the sex of those cells.

**Conclusions:**

The complement of sex chromosomes in cells studied in culture has the potential to affect expression of proteins and 'mechanistic' signaling pathways. Therefore, consistent with scientific excellence, editorial policies should require reporting sex of cells used in *in vitro *experiments.

## Background

In 2001, the Institute of Medicine published a landmark report identifying the importance of sex as a biological variable in experimental and human studies [1]. Over a decade later, the effect of this report remains to be realized, as few clinical trials report outcomes stratified by sex [2,3], and only 22-42% of studies published in journals on neuroscience, endocrinology, physiology, pharmacology, reproductive medicine, and general biology reported the sex of the experimental animals used [4].

Cultured cells are used to identify molecular-signaling pathways. Results from such studies are often used as the basis for development of new therapeutic, diagnostic, and other interventions for translation to human medicine. Because every cell has a sex [1], and the complement of sex chromosomes has the potential to influence expression of proteins and other signaling molecules [5-12], we undertook a literature review to determine how sex was reported for *in vitro *experiments. The review was limited to journals publishing studies in cardiovascular research, because cardiovascular disease remains the foremost cause of mortality for both women and men, and there remains a significant disparity in all-cause mortality from cardiovascular disease between women and men in the USA [13,14]. Some of the disparity in all-cause mortality from cardiovascular disease may result from socioeconomic issues and access to health care. However, other factors contributing to the disparity include sex differences in presentation of symptoms (such as those related to myocardial infarction and stroke [15]), ineffective or adverse response to treatments (side-effects related to angiotensin-converting enzyme inhibitors, treatment for hypertension, or absence of effective treatment for the female-predominant diseases including heart failure with preserved ejection fraction (HFpEF) [14,16]) and presence of cardiovascular diseases unique to women (such as hypertension disorders of pregnancy [17]). The physiological, cellular and molecular bases of how these factors contribute to sex disparity in all-cause mortality remain to be explored.

## Methods

### Search strategy

In order to emphasize how the practice of various editorial committees and policies affect the reporting and transfer of knowledge to the cardiovascular scientific community, the cardiovascular journals with the top impact factors under the subject category 'cardiac and cardiovascular systems' were selected from the ISI Web of Knowledge website under the 2009 JCR Science edition. The list was limited to journals with the top 40 impact factors, and then narrowed in sequential descending order to those journals that published original basic science articles, eliminating journals that published only review articles. Journals were selected in descending order of impact factor: *Circulation; Journal of American College of Cardiology (J Am Coll Cardiol); European Heart Journal (Eur Heart J); Circulation Research (Circ Res); Arteriosclerosis*, *Thrombosis and Vascular Biology (Arterioscler Thromb Vasc Biol); Cardiovascular Research (Cardiovasc Res); Journal of Molecular and Cellular Cardiology (J Mol Cell Cardiol); American Journal of Physiology - Heart and Circulatory Physiology (Am J Physiol Heart Circ Physiol); Journal of Heart and Lung Transplantation (J Heart Lung Transplant*); *and Journal of Cardiovascular Pharmacology (J Cardiovasc Pharmacol*). The journal *Arteriosclerosis*, *Thrombosis and Vascular Biology *was not listed under the subject category mentioned above; however, because of its high impact factor in the cardiovascular field, it was included.

The search terms 'cultured' and 'cells' were used in any order, and were limited to the title, abstract and text, using specific journal websites or, in some cases, the medium used by these journals for their searches, such as Science Direct. The year was limited to '2010' or to the issue(s) that corresponded to the year 2010. Results from these searches then were sorted by best match (relevance). Only original, full-length articles were reviewed; reviews, letters and brief communications were excluded. Two types of searches were conducted.

In the first search, the Methods sections were reviewed for indication of use of cultured cells. Only studies that utilized primary cultures (that is, cells isolated from animals and subsequently cultured) were included. Additionally, we also excluded any articles for which the Methods section was not clear regarding the use of cells isolated from animals for culturing purposes. For each journal, the first 10 articles that satisfied these specifications were included in the analysis. One journal was omitted from the analysis because it did not have 10 articles meeting the inclusion criteria for the year 2010. From this first search, 90 articles meeting our inclusion criteria were identified.

In the second search, the top 10 journals were searched again using the terms 'culture' and 'cells' in any order for best match (relevance), but the supplemental material was also reviewed and articles were limited to those using either endothelial cells (ECs), vascular smooth-muscle cells (VSMCs), cardiomyocytes (including neonatal cardiomyocytes), and cardiac fibroblasts (including neonatal cardiac fibroblasts), as well as stem cells with a clear indication of differentiation into a cardiac lineage. Additionally, the number of articles from two randomly selected journals (*Cardiovasc Res *and *J Mol Cell Cardiol*), were increased to 20, in order to reduce potential sampling bias. As a result of this second strategy, 101 articles were reviewed, but the number of articles per journal varied.

#### Data collection

Data were collected on the sex of cells, scored as 'yes' for sex reported and 'no' for no sex reported. The 'yes' was further categorized into males, females, or both. Additionally, the species from which the cells were isolated and the type of cells used were recorded.

## Results

From this survey of the top 10 cardiovascular journals based on impact factor, we found that reporting the sex of the cells used in experiments is not standard. Using the first search strategy, we found that only 25 articles (28%) of the 90 articles surveyed reported the sex of cells used in the experiments.

**Figure 1:**
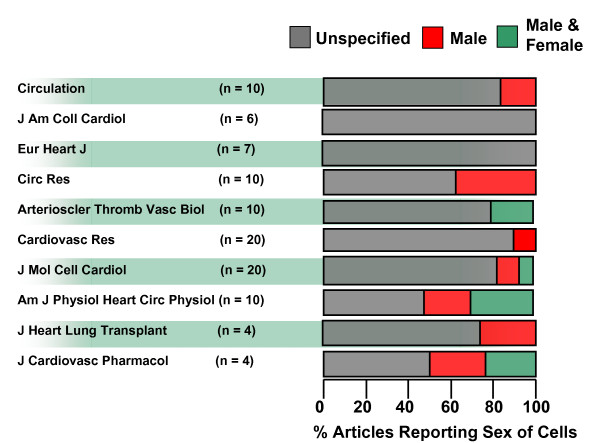
**Percentage of articles reporting sex of cells used in the experiments**. Percentage of articles reporting sex of cells used in the experiments. n = Number of articles reviewed per journal. None of the studies reviewed used only cells from female animals.

Using the second search strategy, we identified 101 articles from 10 journals; only 20 of these articles (19.8%) reported the sex of the cells used in the experiments (Figure 1). Of those articles that reported the sex of cells, 85% did so within the body of the article, whereas 15% of articles reported it within 'supplemental material.' The numbers also varied between journals: half of the articles surveyed from *Am J Physiol Heart Circ Physiol and J Cardiovasc Pharmacol *reported the sex of cells used in the experiments, compared with none of the articles in this survey from *Eur Heart J and J Am Coll Cardiol*. Reporting of sex in the other journals reviewed ranged from 10% to 40% of the total (Figure 1).

Of the 20 articles from the second search in which sex was reported, male cells predominated, and were used in 65% of these articles, whereas cells from both sexes were used in only 35%. None of the studies used exclusively female cells. For instance in *Am J Physiol Heart and Circ Physiol*, two of the five articles reporting the sex of the cells used cells derived from male animals; the remaining three studies used cells from both sexes. By contrast, studies published in *Cardiovasc Res*, the journal with the lowest percentage of sex reporting, used cells from male animals only.

**Figure 2:**
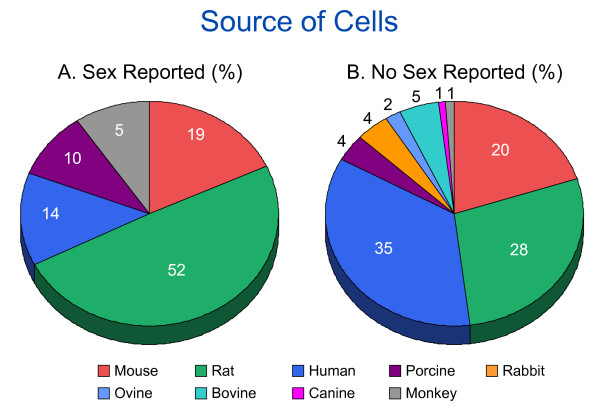
**Representation of the species for source of cells**. Representation of the species for source of cells (by percentage) which **(A) **reported (n = 20 articles) and **(B) **did not report (n = 81) the sex of cells in 101 articles reviewed by the second search strategy. Some studies used cells from more than one species. **(A) **In studies that reported the sex of the cells, most used cells derived from rats, whereas **(B) **in studies that did not report sex, most cells were derived from humans.

The species of the cells were reported in all studies (n = 191), even those that did not report sex. The cell types reported were: human, rodent (rats and mice), bovine, porcine, ovine, rabbit, primates and canine. The sex of cells derived from rats was reported most often whereas the sex of cells derived from humans was reported least often (Figure 2).

**Figure 3:**
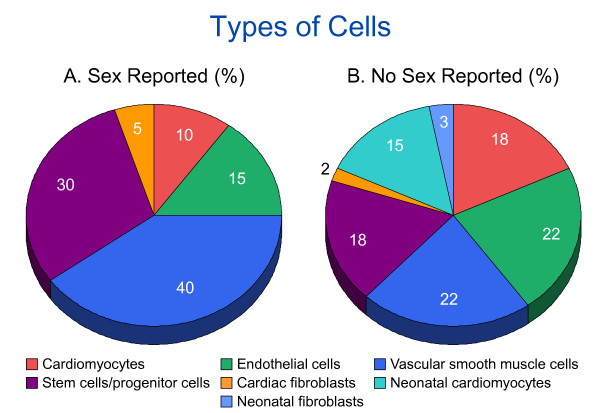
**Representation of cell types**. Representation of cell types (by percentage) which **(A) **reported (n = 20 articles) and **(B) **did not report (n = 81) the sex of cells in 101 articles reviewed by the second search strategy. Some studies used more than one type of cell. **(A) **In studies reporting the sex of the cells, most used vascular smooth-muscle cells, whereas in studies that did not report the sex of cells, most used endothelial cells and vascular smooth-muscle cells.

None of the articles that reported the sex of the cells had used neonatal cardiomyocytes. ECs and VSMCs were the cell types used most often in studies that did not report sex, and VSMCs were those used most often in the articles that did report the sex of cells (Figure 3).

## Discussion

The results of this literature review indicate that in cardiovascular specialty journals with high impact factors, sex, despite being a biological variable that could affect outcomes, interpretation and applicability of data, is not reported consistently. This observation parallels the absence of reporting of sex found in journals and articles reporting results of studies using animals and of human clinical trials [2-4]. Unlike the analysis of articles using experimental animals, which covered a variety of disciplines, the present search reviewed only articles reported in journals with a cardiovascular focus. Therefore, it is not possible to extrapolate these findings to journals that publish articles using cultured cells in other disciplines, or to the use of other search strategies with different inclusion/exclusion criteria for sampling. However, this sample included a variety of journals, representing both those published by for-profit publishers and by not-for-profit entities (such as professional societies), and those with international editorial boards. Because the same results were obtained from one journal reviewed by two independent researchers using different strategies (as an internal control), it is unlikely that our results were biased by reviewer or strategy.

In the United States, Canada and the European Union, regulatory mandates require inclusion of women in clinical trials. Similar mandates do not exist for preclinical or basic studies. Because studies using cells in culture are considered a first step in the identification of mechanistic and molecular approaches for drug discovery, it is important to identify those pathways and conditions for which the sex of the cell may influence gene expression and potential therapeutic molecular targets. Based on the results of this review, there seems to be an assumption that the sex of cells in culture is not important or is a detail that does not warrant reporting. This assumption is false, given the evidence that sex influences expression of genes in some cultured cells, tissues, organs and diseases, including cardiovascular disease [7,8,12,13,18-20]. In 2001, the General Accounting Office reported that pharmaceuticals withdrawn from the market in the USA had greater health risks for women than men (GAO-01-286R). Evaluations have not been performed on pharmaceuticals withdrawn from the market since the dates covered in the 2001 report. However, the possibility exists that identification of sex differences in molecular targets and cellular responses in the initial, often less expensive, stages of drug development might identify new targets that could reduce adverse side-effects in clinical testing. Indeed, adverse drug events as collected by the Italian Agency of Drugs have been found to be greater in women than men (2010 statistics). In 2011, the Italian Medicines Agency (AIFA) established a Working Group on Medicines and Gender for the purpose of studying issues related to regulatory and pharmacological issues in gender medicine.

In this era of genomics, the concept that the complement of sex chromosomes might influence outcomes/results of experimental studies using cultured cells seems fundamental. Journals do not have specific policies requiring reporting of the sex of cells, even though other experimental details (such as culture conditions of temperature, oxygen tension and media) are reported (Table 1). It is likely that researchers, reviewers and editors simply do not think about sex as an important biological variable in the experimental design [21]. There is often the assumption and rationale that inclusion of female material would create additional variation in outcomes relative to male material. This assumption warrants questioning, as does the procedure of culturing male cells in media such as fetal calf serum, which contains large amounts of female sex-steroid hormones unless stripped in charcoal. Material from neonatal animals is studied to understand factors contributing to cellular differentiation; in this survey, none of the studies using cardiac myocytes or fibroblasts from neonatal animals reported the sex of the neonates even though it is possible to sex these animals using anogenital distance [22]. The use of cultures of cells of mixed sex assumes equal viability, function and differentiation for cells of male and female animals. This assumption requires validation, as genes on the Y chromosome may affect risk for cardiovascular disease [23-25], and genes on the X chromosome may affect responses to hypoxia and apoptosis, processes involved in cardiac performance [9-11,26,27].

**Table 1:** 'Instruction to Authors' and editorial policy for journals from which reviewed articles were obtained.

Journal	Publisher	Policy
*Circulation*	American Heart Association	'Experimental animals: State the species, strain, number used and pertinent descriptive characteristics.'

*J Am Coll Cardiol*	Official Journal of the American College of Cardiology/Elsevier	'Manuscript submissions should conform to the guidelines set forth in the "Uniform Requirements for Manuscripts Submitted Biomedical Journals: Writing and Editing for Biomedical Publication"... Animal investigations must conform to the "Position of the American Heart Association on Research Animal Use"'

*Eur Heart J*	Official Journal of European Society of Cardiology/Oxford Journals	No specific information in Instruction to Authors

*Circ Res *	American Heart Association	'Expanded Methods section must... be detailed enough to enable readers to replicate the experiments without consulting previous articles... For animals used in experiments, state the species, strain, number used and other pertinent descriptive characteristics.'

*Arterioscler Thromb Vasc Biol*	American Heart Association	'...state the species, strain, number used and other pertinent descriptive characteristics.'

*Cardiovasc Res*	Oxford Journals	'....procedures with animals or animal tissues, investigations conform to either the Guide for the Care and Use of Laboratory Animals... or the Directive 2010/63/EU.'

*J Mol Cell Cardiol*	Official Journal of the International Society for Heart Research/Elsevier	'the use of animals...the authors should ensure that the manuscript contains a statement that procedures were performed in compliance with relevant laws and institutional guidelines... '

*Am J Physiol Heart Circ Physiol*	American Physiological Society	'Describe techniques, cell/animal models used'

*J Heart Lung Transplant*	Elsevier	'All manuscripts reporting experiments using animals must include a statement in the Methods section giving assurance that all animals received humane care in compliance with the "Principles of Laboratory Animal Care" formulated by the National Society for Medical Research and the "Guide for the Care and Use of Laboratory Animals" prepared by the Institute of Laboratory Animal Resources and published by the National Institutes of Health (NIH Publication No.86-23, revised 1996).'

*J Cardiovasc Pharmacol*	Wolters Kluwer/Lippincott/ Williams & Wilkins	'... studies on experimental animals have been conducted under protocols reviewed and approved by the author's institutional animal care and use committee and to adhere to generally accepted international guidelines for animal experimentation.'

Although this survey focused specifically on reporting of the sex of cultured cells, it should be mentioned that it is possible that the gene expression of these cells may be set or influenced by the hormonal environment of the animal from which the cells were derived. For example, long-lasting effects of hormones on cellular function have been explored in other systems [28-30]. These issues have yet to be considered in studies of cardiovascular cells. Thus, for example, it remains to be determined whether cultured human umbilical-vein ECs are representative of ECs derived from an adult or aged male or from a null- or multi-parous female. As indicated in the 2001 Institute of Medicine report [1], sex, age and hormonal status need to be considered in experimental design.

The editors of scientific journals and the publication committees of professional societies are in the position to direct change for a broader inquiry into sex-driven mechanisms of disease by requiring reporting of the sex of the experimental material used and the analysis of data by sex [21]. Authors are accustomed to having to include in their Methods sections statements of institutional approval for use of animals or humans, funding sources and conflict of interest. Although those items are important to insure the objectivity of and regulations for the conduct of the study, they are unlikely to directly affect the results of the experiments or to allow others to replicate their experiments, a traditional requirement for scientific validity. Perhaps it is time to require reporting of the sex of the material used in studies. Not only might sex affect experimental outcome, but reporting such a fundamental component of the experimental design is consistent with scientific excellence.

## Conclusion

Sex is not consistently reported as a variable in studies using cultured cells to investigate mechanisms of cardiovascular physiology and disease. The editorial policies of scientific journals should require reporting of sex as a genetic variable and as a fundamental condition of scientific excellence.

## Competing interests

This review was performed as part of a course assignment for Advanced Cardiovascular Seminar BME 8855, Graduate School of Medicine, Mayo Clinic. Dr. Miller is course director, President of the Organization for the Study of Sex Differences (2010 - 2012) and a member of the Board of Directors for the Society for Women's Health Research.

## Authors' contributions

KET and CV-G acted as joint team leaders for data collection and analysis, and contributed to writing all sections of the paper. NSS and RZ reviewed the journal articles for data collection. VMM acted as mentor for study design, data analysis and validation, and contributed to writing and editing all sections of the paper. All authors read and approved the final version of the manuscript.
